# Myelin in Alzheimer’s disease: culprit or bystander?

**DOI:** 10.1186/s40478-023-01554-5

**Published:** 2023-03-31

**Authors:** Michel Maitre, Hélène Jeltsch-David, Nwife Getrude Okechukwu, Christian Klein, Christine Patte-Mensah, Ayikoe-Guy Mensah-Nyagan

**Affiliations:** 1 Biopathologie de la Myéline, Neuroprotection et Stratégies Thérapeutiques, Fédération de Médecine Translationnelle de Strasbourg (FMTS), INSERM U1119, Université de Strasbourg, Bâtiment CRBS de la Faculté de Médecine, 1 rue Eugène Boeckel, Strasbourg, 67000 France; 2 Biotechnologie et signalisation cellulaire, UMR 7242 CNRS, Université de Strasbourg, 300 Boulevard Sébastien Brant CS 10413, Illkirch cedex, 67412 France

**Keywords:** Myelin, Early biomarker, Alzheimer’s disease, Oligodendrocytes, Aβ peptides

## Abstract

Alzheimer’s disease (AD) is a neurodegenerative disorder with neuronal and synaptic losses due to the accumulation of toxic amyloid β (Αβ) peptide oligomers, plaques, and tangles containing tau (tubulin-associated unit) protein. While familial AD is caused by specific mutations, the sporadic disease is more common and appears to result from a complex chronic brain neuroinflammation with mitochondriopathies, inducing free radicals’ accumulation. In aged brain, mutations in DNA and several unfolded proteins participate in a chronic amyloidosis response with a toxic effect on myelin sheath and axons, leading to cognitive deficits and dementia. Αβ peptides are the most frequent form of toxic amyloid oligomers. Accumulations of misfolded proteins during several years alters different metabolic mechanisms, induce chronic inflammatory and immune responses with toxic consequences on neuronal cells. Myelin composition and architecture may appear to be an early target for the toxic activity of Aβ peptides and others hydrophobic misfolded proteins. In this work, we describe the possible role of early myelin alterations in the genesis of neuronal alterations and the onset of symptomatology. We propose that some pathophysiological and clinical forms of the disease may arise from structural and metabolic disorders in the processes of myelination/demyelination of brain regions where the accumulation of non-functional toxic proteins is important. In these forms, the primacy of the deleterious role of amyloid peptides would be a matter of questioning and the initiating role of neuropathology would be primarily the fact of dysmyelination.

## Introduction

Sporadic AD typically occurs after the age of 65 and is the most common cause of dementia in older people. We consider here the disease under its main pathophysiological definition that classically consists of extracellular amyloid plaques and intracellular neurofibrillary tangles [69, 70]. These abnormalities lead to a cascade of events eventually conducting to cognitive disorders and dementia. There are, however, a significant number of diverse clinical presentations with ages of onset and evolution in the disease that suggest non-univocal pathophysiological mechanisms [61]. It seems that some cases of sporadic AD involve changes in the constitution and architecture of myelin, and this early in the life of the future patient. In general, extracellular abnormalities of the amyloid cascade predominate, followed most often by other Tau mediated biological mechanisms at the intracellular level, accompanied by inflammatory, neuroimmune and neurochemical disorders that can put dysmyelination at the forefront of neurodegenerative disorders.

**Fig. 1:**
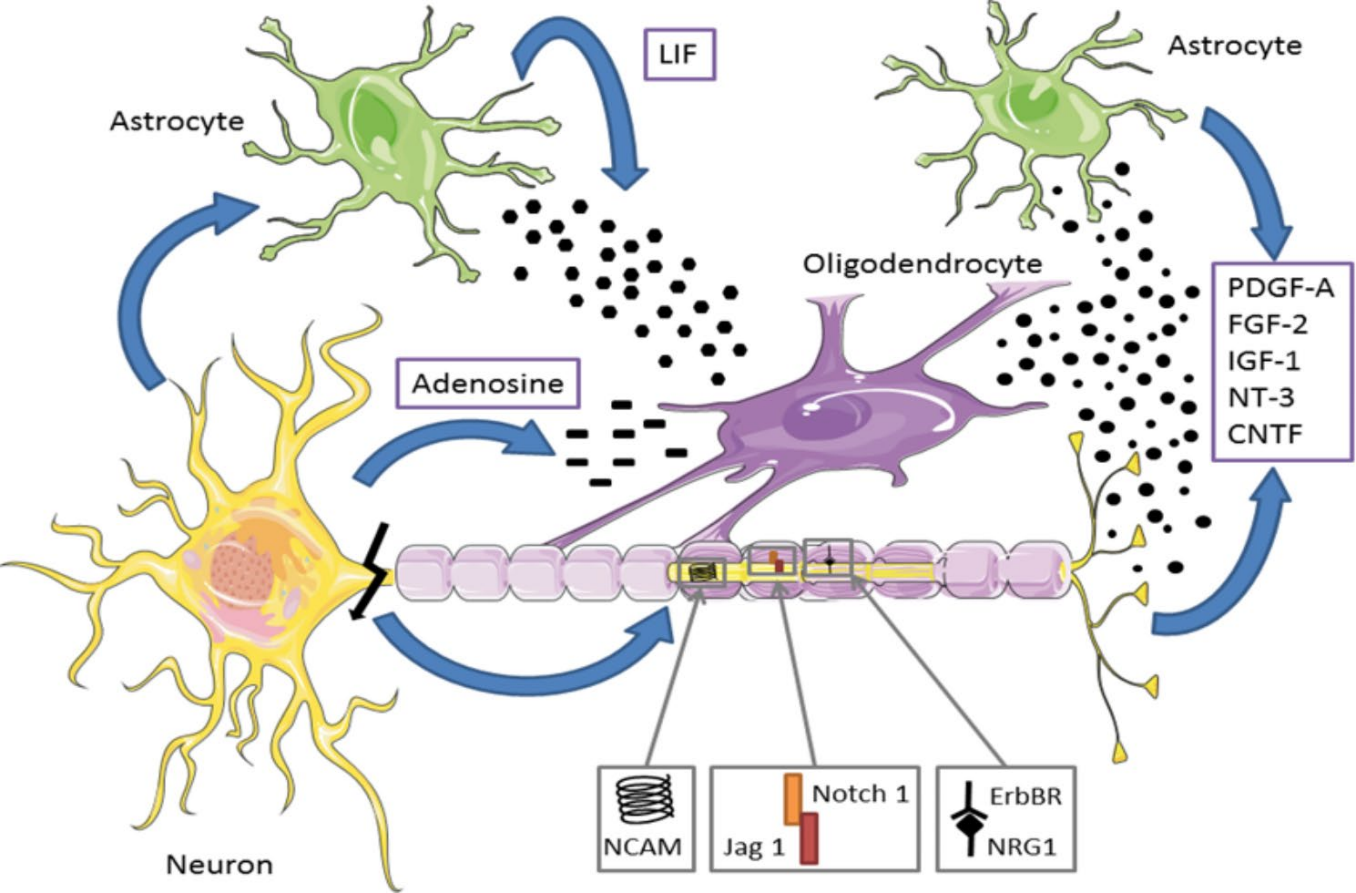
**Oligodendrocytes are derived from the differentiation of oligodendrocyte precursor cells (OPCs) and are the main cell for remyelination. ** (modified from [175]). The differentiated oligodendrocytes of OPCs migrate to different axons via positive chemotactism [216]. A variety of growth and trophic factors regulate the development of oligodendrocytes and their temporal and geographical attractions [17]. Many of these factors are produced by both neurons and astrocytes, regulating the proliferation, survival, or degeneration of OPCs. The neuroregulin, which activates Erb-tyrosines kinases receptors, promotes the survival and proliferation of oligodendrocytes. The activation of the Notch 1 cascade inhibits the differentiation of oligodendrocytes, and an integrin/contactin complex coordinates signals from the extracellular matrix and the axonal surface to regulate oligodendrocyte survival and myelination. This also depends closely on the electrical activity propagated in the axons. OPCs express functional adenosine receptors, activated by action potential [149, 183]. Adenosine acts as a powerful transmitter between glia and neurons to inhibit the proliferation of OPCs, stimulate their differentiation and stimulate myelin production. The LIF (leukemia inhibitory factor) is heavily involved in oligodendrocyte development kinetics and in the overall myelination process [133, 198]. ***Abbreviations***: *CNTF, ciliary neurotrophic factor; FGF, fibroblast growth factor; IGF, insulin-like growth factor; LIF, leukemia inhibitory factor; NCAM, neural cell adhesion molecule; NT-3, neurotrophin 3; OPCs, oligodendrocyte precursor cells; PDGF-A, platelet-derived growth factor-A*

Myelin consists of a multilayered membrane wrapped around the axons of most central nervous system (CNS) neurons. This membrane is produced by expansions of specialized glial cells of the brain, “the mature oligodendrocytes”, derived from oligodendrocytes progenitor cells (OPCs) [21]. This cell line constitutes the precursor cells for the constitution of the myelin sheath through a well-defined program of proliferation, migration, and differentiation to lead to the myelination of neuronal axons [100]. Among the properties of myelin, the best known is the saltatory conduction of nerve impulses, which gives it more speed and efficiency. Furthermore, it is now well acknowledged that oligodendrocytes’ expansions display a trophic, plastic, and metabolic influence on the axons they envelop (Fig. 1) [130, 139]. Myelin is constantly reshaping and its alteration in degenerative phenomena such as Alzheimer’s disease (AD) may be a fundamental element for the genesis of pathophysiological and clinical disorders observed in the early stages of the disease [22].

AD involves progressive neurodegeneration with neuronal losses leading to cognitive, memory, emotional, behavioral disorders, and a progressive dependence [210]. Long considered to primarily affect the grey matter, many studies have described early lesions of the white matter in patients with nascent and moderate intensity [55]. The main molecular alterations of the disease are considered to contain essentially a pathology of the production/degradation and an intra-brain accumulation of amyloid β (Aβ) peptides producing deposits in the form of hydrophobic plates of aggregated toxic peptides (senile plaques) [69, 159]. Another proteinopathy usually accompanies the previous protein in the form of hyperphosphorylated tau proteins and deposits of neurofibrillary tangles. These toxic proteins maintain chronic inflammation and oxidative stress accompanied by neuronal and synaptic losses at the origin of the symptomatology [31]. This paper reviews the fundamental importance of myelin and correct CNS myelination for its development, functional adaptations, and permanent reshuffling. During aging in the human patient, sporadic AD has plurifocal impairments that induces various clinical presentations depending on the intensity of inflammatory and immune reactions, and ischemic, mitochondrial, and free radical disorders. In many cases, the hypothesis of an alteration of the amyloid cascade Aβ as a primitive mechanistic etiology is questionable and multiple proteinopathies can be implicated, which depend on somatic mosaicism, transcriptional and translational alterations. In many cases, myelin and its integrity appear to be a preferential and early target in multiple forms of AD, and oligodendrocytes represent a cell population highly sensitive to Aβ and other proteinopathies. These misfolded proteins result in multiple dysmetabolism that accentuate and modify the course and clinical forms of the disease.

## Multiple causes, multiple targets

### AD as an heterogeneous disease

The amyloid hypothesis as the essential cause of neuronal loss and brain atrophy is a matter of discussion mainly because removal or reduction of amyloid plaques by immunological treatments display no significant effect on clinical symptoms of AD [39, 140]. However, before aggregation, the soluble oligomeric forms of Aβ peptides possess strong toxic properties against myelin integrity and neuronal survival [160, 188]. White matter lesions are commonly found in magnetic resonance imaging (MRI) scans of elderly people and are associated with cognitive decline [128]. Whether or not a primary role of Aβ peptides is fundamental in these lesions is a matter of debate.

Increase in the concentration of Aβ peptides in brain has also been described because of head trauma or cerebral ischemia [178, 220]. This increase in the concentration of Aβ peptides is additional evidence for the existence of various forms of Alzheimer’s like diseases with various clinical pictures and various mechanisms of neurodegenerative processes in term of pathophysiological evolution and biological markers [43, 214]. In addition to Aβ peptides accumulation and toxicity, hyperphosphorylation of tau proteins, which disturbs microtubules assembly and axonal transport, could have some impact on the trophic effect on the myelin envelop [209]. These basic alterations are also supplemented with several others biological modifications of many molecular pathways and functions namely in the domain of energy metabolism and cholesterol transports [19]. Cholesterol is fundamental for oligodendrocytes survival and for synthetis of myelin, this compounds represent a large proportion of the human brain and abnormalities in cholesterol metabolism are present and associated with brain age and in Alzheimer’s disease [12, 105]. Regarding cholesterol delivery to axons and synapses, the ε4 allele of the apolipoprotein E (*APOE*) gene is the less effective factor for cholesterol transport compared to ε3 and ε2. Interestingly, normal adults at the cognitive level show microstructural changes in myelin architecture when carrying homozygous alleles ε4 that is a major marker for late AD [128, 145, 146].

### Sporadic AD is a multifactorial disease

While familial AD has essentially genetic causes expressed in amyloid precursor protein (APP) and presenilin leading early in life to specific proteinopathies and inducing neurodegenerative pathologies [63], these mechanisms are less consistent in the sporadic AD of elderly subjects. In these patients displaying accumulations of cerebral amyloid peptides, the question arises as to whether this accumulation is the cause or the consequence of other factors inducing neurodegeneration. Over the years, many mutations are present in brain neurons, generating multiple proteinopathies after transcriptional, translational, or post-translational errors. Some of these are of exogenous origin and enter the CNS due to the pathological porosity of the blood-brain barrier (BBB). The accumulation of these abnormal proteins generates inflammatory and immune responses that lasts for many years. Such accumulation is increased by mitochondriopathies, and the genesis of free radicals related to the disruption of oxidative phosphorylation and the depreciation of energy metabolism. The most deleterious and widespread proteinopathy is that affecting the regulated proteolysis of the APP, giving rise to toxic peptides interfering with many neuronal functions and leading to synaptic and neuronal losses, as well as inducing profound cognitive and behavioral functional abnormalities. These peptides exhibit amyloid properties and accumulate over time into hydrophobic plaques, which can be detected by PET scan ligands or post-mortem histology.

Accumulating evidence supports a multi-factor for the origin of many forms of sporadic AD. Multi-organ alterations could initiate or worsen neurodegeneration [8, 193, 208]. Developing heart failure promoting hypoxia, intestinal and hepatic disorders altering brain metabolism through the microbiome, ischemic symptoms due to vascular deposits and chronic inflammation, could also contribute to the decrease in neuronal survival [114, 197].

### Metabolic disorders and AD

**Fig. 2:**
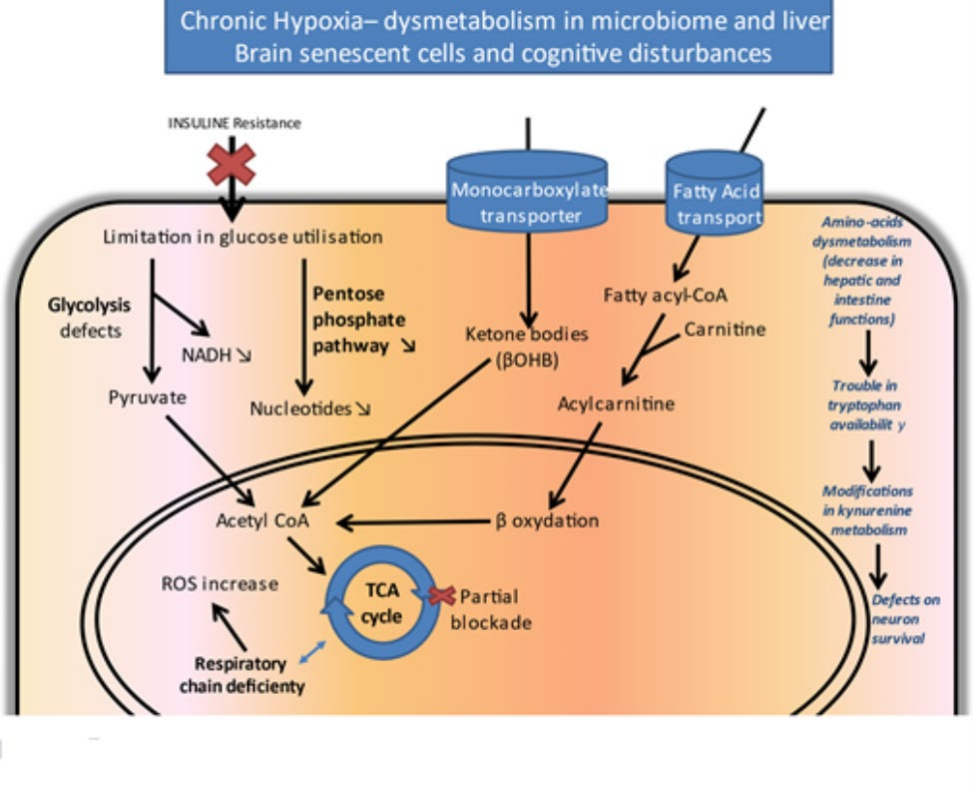
**Dysregulation in multiple biochemical pathways underlie the pathogenesis of AD.** Metabolomic approaches conducted from the blood or CSF of AD patients compared to controls highlighted abnormalities in the energy metabolism of patients. A diabetic-type pathology is often evoked with a decrease in insulin sensitivity. In addition to disorders in glycolysis and the respiratory chain, abnormalities involving accumulations of ketone bodies resulting from the metabolism of acetyl-CoA residues, a product of the accelerated degradation of fatty acids by β-oxidation, have been described. The bioavailability and metabolism of several amino acids could also be affected, especially concerning tryptophan degraded in the kynurenine cycle and resulting in the formation of neuroprotective (kynurenic acid) or neurotoxic (quinolinic acid) compounds. ***Abbreviations***: *AD, Alzheimer’s disease; CSF, cerebrospinal fluid; NADH, nicotinamide-adenine-dinucleotide; ROS, reactive oxygen species; TCA, tricarboxylic acid; βOHD, beta-hydroxybutyrate*

Many metabolic alterations have often been encountered in AD with varying severities. Most of these alterations concern or affect brain energy metabolism, these phenomena being aggravated by cerebral hypoperfusion and blood sugar abnormalities [10]. Carbohydrate metabolism and dysfunctions in intestinal absorption phenomena, nutritional abnormalities and deficiencies, resistance to glucose utilization via decreased insulin sensitivities combined with fatty acid metabolism disorders, induce energy deficiencies deleterious to neuronal functioning and survival [85]. The slowing down of the tricarboxylic cycle generates the accumulation of acetyl-CoA coming from the increased fatty acids degradation and the synthesis of ketone bodies that could have a positive role on neuronal survival. White matter degeneration in AD could be in part due to the accelerated degradation of lipids in this context of decreased energetic metabolism coming from reduced glucose utilization (Fig. 2) [141, 206].

At the hepatic level, functional alterations (cirrhosis, hepatitis) could aggravate the elimination of deleterious proteins including Aβ [201]. Modifications of bile acids synthesized by the altered liver tissue exhibit impaired neuroprotective functions. Frequently, the accumulation of mutations in the mitochondrial genome accelerates pathological phenomena at the level of the tricarboxylic cycle or the respiratory chain and increase ROS production [73]. Metabolic disorders of the periphery of the body often affect brain metabolism via abnormal permeability of BBB and the presence of abnormal metabolites in the cerebrospinal fluid (CSF). This mainly concerns certain intermediates of amino acids metabolism, particularly regarding the catabolites of tryptophan degradation [167]. This essential amino acid is the precursor not only of melatonin and serotonin, but also of the intermediates of the kynurenine cycle, some of which possess neuroprotective or neurotoxic properties or interfere with the elimination of amyloid peptides from the brain [116].

## Myelin has morphological alterations in the early stages of AD

### Change of the lipid composition of myelin over time [127]

**Fig. 3:**
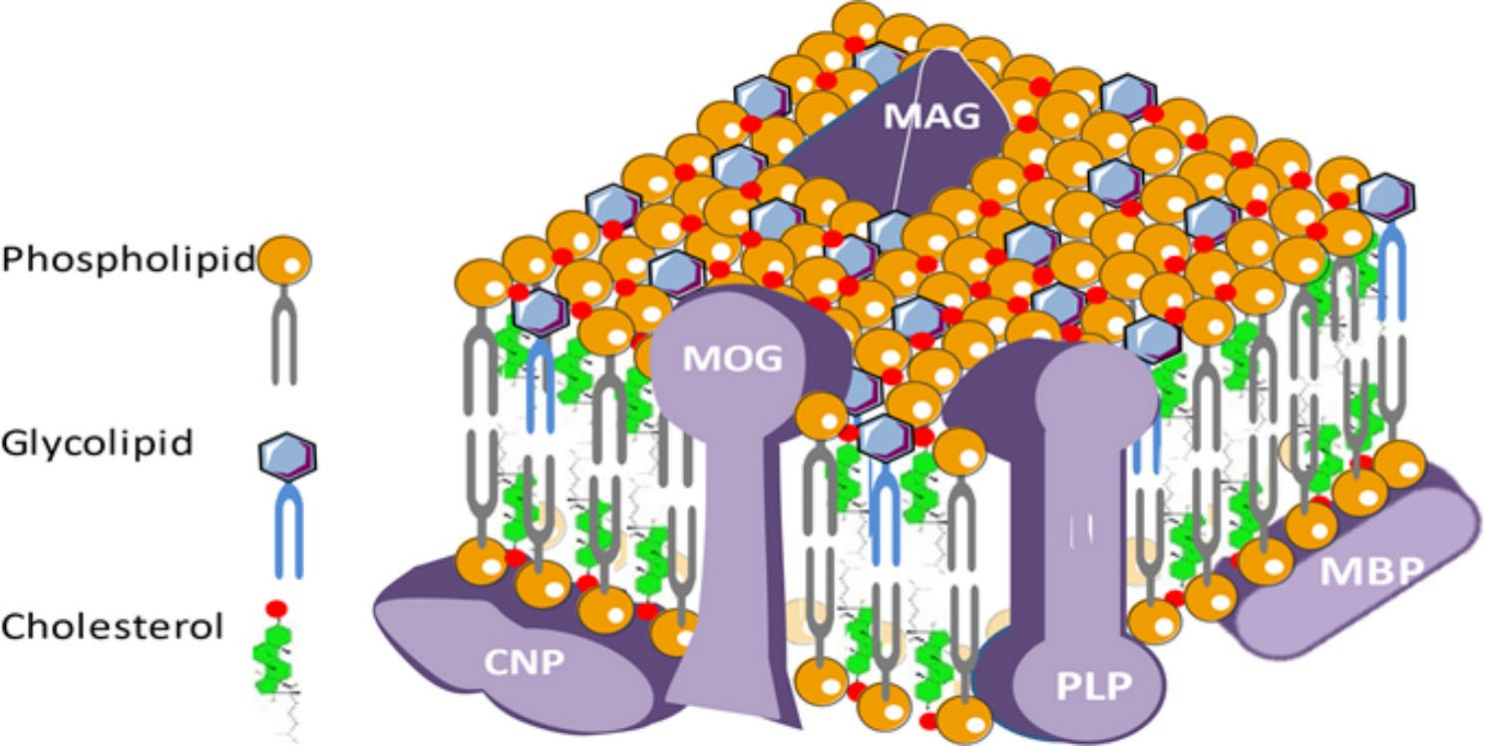
**Myelin composition and organization.** The myelin wrapping around most of the CNS axons includes a large majority of complex lipids and 15–30% of specific proteins. Lipids are essentially made up of cholesterol, galactocerebrosides and phospholipids. This envelope is constantly reshuffled in time and space from the oligodendrocytes that make up the bulk of the glial cells of the CNS. Chronic inflammatory and autoimmune reactions, mutations in certain constituent proteins, attacks by free radicals or ischemic, and metabolic problems related to aging alter the myelin sheath that releases its constituents into the CSF and the bloodstream. In AD, myelin is one of the first bulwarks for the anatomical and functional integrity of the axons it surrounds and undergoes early toxic action of misfolded extracellular toxic proteins or peptides. ***Abbreviations***: *AD, Alzheimer’ disease; CNP, C-type natriuretic peptide; CNS, central nervous system; CSF, cerebrospinal fluid; MAG, myelin-associated glycoprotein; MBP, myelin basic protein; MOG, myelin oligodendrocyte glycoprotein; PLP, proteolipid protein*

Oligodendrocytes-derived myelin accounts for about 40% of CNS lipids, consisting of 50% phospholipids, 40% glycolipids, 10% cholesterol and cholesterol esters, and polyunsaturated long-chain fatty acids (Fig. 3) [82] .

Synthesized by oligodendrocytes, cholesterol comes almost exclusively from ketone bodies as precursors. This lipid has structural functions at the level of the myelin by regulating the fluidity and permeability of this membrane around the axons, and it regulates the speed of myelination according to its uptake by the membrane in formation. The typical lipids of myelin are essentially galactosyl ceramides and sulfatides. They stabilize and organize myelin in direct association with the basic protein.

Changes in the configuration of myelin are observed with age but are more accentuated in AD. Not all regions of the brain are affected in the same way. In general, the volume of white matter decreases over time and the phenomena of demyelination/remyelination accentuated by pathology leads to the decrease in the size of axons and the reduction in the size of the internodal distances. These structural changes induce functional consequences for conduction rates and vulnerability to traumatic, ischemic, dysmetabolic conditions and toxic factors such as oligomers Aβ peptides. These processes are commonly encountered as factors favoring Alzheimer’s genesis and pathology.

### A step for the conversion of mild cognitive impairment (MCI) into dementia?

Studies of myelin sheath’s conformation in AD were mostly conducted by electron microscopy and MRI both in animals and humans. The 5XFAD mouse is a transgenic model that expresses three different mutations in the *APP* and two in presinilin 1 (*PS1*). In this mouse, amyloid deposits can be detected with synaptic losses at an age as early as 1.5 months [142] and myelin abnormalities can be seen even earlier accompanying the first alterations in spatial memory occurring around the age of 1 month [67]. Several studies conducted in humans suggest that myelin disorders strongly contribute to the onset of AD symptoms. Neuroimaging shows myelination defects in several brain regions, but especially and firstly in the hippocampus and corpus callosum [55, 137, 147, 202]. Conformation abnormalities accompanied by thinning of the myelin sheath are frequently encountered even before the onset of axonal lesions, which may indicate premises for demyelination. In the pre-clinical stages of the disease, MRI shows altered longitudinal and transverse relaxation times and increased myelin hydration degrees [18]. In general, abnormalities in the structure and formation of the cerebral white matter have been identified in many presentations of Alzheimer’s disease that can be warning signs for a disease in progress [156]. Variations in T1w/T2w ratios in patients with risk factors (close family history, *APOE4* phenotypes) were identified compared to control individuals. In addition, individuals at risk had an association with altered patterns of resting-state functional connectivity (rs-FC) [52]. These abnormalities support the idea of significant alterations in myelin developing with age and constituting signals of vulnerability [53]. Interestingly, some studies have shown a relationship between structural abnormalities of myelin in ADs in the pre-clinical period and peptide concentrations of Aβ1–42 in patients’ CSF [34, 38].

Studies of cortical stratification in the human brain provide important knowledge on the level of degeneration, in addition to the information given by the level of volumetric atrophy [147, 155]. MRI studies show hyper-densities in the white matter with volume increase consistent with the abnormalities of amyloid peptides and tau proteins in CSF. At the histological level, it appears that these stratification disorders are mainly due to alterations in myelin architecture in which iron ions could play an important role [194]. Vascularization and oxygen supply in injured hyper-dense regions are decreased and are related to axonal lesions and inflammatory disorders, BBB permeability abnormalities and multiple disseminated micro-hemorrhagic structures [88, 102].

## Potential involvements of epigenetic mechanisms in myelin reshuffle

GWAS (genome-wide association study) has identified about 40 loci associated with AD in the European population and in these respective loci, several genes involved directly in the causative mechanism of the disease have been described (*APOE, CR1, BIN1, TREM2, CLU SORL1, ADAM10, ABCA7, CD33, SP11, PIRLA*). It remains to identify the functions of many genes in the identified loci. Many risk genes are involved in the innate immune response and neuroinflammation. The CD33 and TREM2 microglia receptors, implicated in microglial pathology, represent new targets for the development of therapeutic tools. It is possible in many cases that the activation of innate immunity, like that encountered in other myelin pathologies, associated with long-term inflammatory mechanisms, is responsible for subtle alterations of myelin during the incubation period of the disease [5, 65].

Over 20 AD risk loci falling mainly in noncoding regions of the genome have been identified by genome-wide association studies, explaining the complexity of the disease at the genetic level [81, 98, 126]. The regulation of gene expression by microRNA is a promising issue for the diagnostic and treatment of several kind of MCI and AD at the beginning of the symptoms, as well as to discriminate with other myelin pathologies like multiple sclerosis [103, 226]. In such diseases, the dynamics of the myelination/demyelination/remyelination balance is continuously evolving under normal conditions of plasticity of the nervous system, but also under pathological conditions, where this balance is affected [47]. In this respect, the process of myelin degeneration are particularly concerned both in multiple sclerosis and in the early phases of AD. Oligodendrocytes and their progenitors are directly involved in membrane and metabolic interactions with neurons during the different phases of destruction and regeneration of the myelin sheath, driven by the dynamic and fluctuating expression of many transcription factors [179]. The activity of the nervous system is intimately linked to the epigenetic regulation of the activity of these factors and to the neo-expression of certain genes involved in the functional dynamics of the production/destruction of myelin [158]. Correct myelination is essential for the proper development and evolution of neuronal connections and the adaptation of brain function to the environment. It constantly reshapes neuron/oligodendrocytes interactions following many factors such as learning, social relationships, emotional stimuli (emotions, anxiety) [166, 217]. These stimuli can induce epigenetic modifications that alter the physiology and functionality of precursors and oligodendrocytes [165].

Changes in the epigenome have a role in the manifestations of AD [13]. Social isolation impacts the intensity of neuronal activity and reduces the importance of myelination [6, 139]. Modifications in the acetylation and methylation of histones were detected, as well as in DNA [131]. These adaptations participate in the regulation of genes involved in the processes of myelination/demyelination and in the pathophysiology of certain neurodegenerative diseases where these processes play a central role (AD, but also multiple sclerosis) [30]. This last pathology of myelin includes analogies with those existing in some phenotypic form of AD and may be the consequence of a combined alteration of genetic and epigenetic factors, the latter involving DNA methylations, histone modifications, chromatin remodeling and modified regulation of non-coding RNA [16].

## Brain markers of myelin in Alzheimer’s patients

### Looking for myelin components in biological fluids

At the genetic markers level, several genes associated with the corpus of the oligodendrocyte ecosystem have been described as risk factors in late-onset AD [124]. In genomic association studies, the *BIN1* (bridging integrator 1) gene is considered to be significantly involved in late AD behind the *APOE* gene [169]. It is mainly expressed in mature oligodendrocytes and white matter in rodents and humans, where it regulates membrane dynamics in the phenomena of endocytosis and membrane remodeling [37]. Histologically, *BIN1* is mostly expressed at the Ranvier nodes.

*BACE1* (beta-site amyloid precursor protein cleaving enzyme 1) codes for a transmembrane β secretase expressed in several cell types including oligodendrocytes. It cleaves *APP* giving birth to amyloid peptides, but also neuroregulin 1, which modulates the myelination and differentiation of oligodendrocytes [50, 195]. Many β-secretase inhibitors have effects on myelin abnormalities caused by AD. Finally, several other genes that are also expressed in oligodendrocytes (*PICALM, NME8, PSEN*, for example) possess a special responsibility as genetic factors associated with the development of AD [123].

*LINGO 1* (leucine rich repeat and Immunoglobulin-like domain-containing protein 1) codes for a transmembrane protein primarily expressed in the cortex, hippocampus, thalamus, and amygdala. The protein acts primarily as a negative regulator of myelination and its inhibition may have potential applications for the treatment of myelin damage in neurodegenerative diseases [204]. As such, anti-LINGO 1 antibodies promote the action of oligodendrocytes and the repair of myelin disease [212].

The biochemical markers of the white matter indicating the evolution of late AD are of many natures and depend on the stage of the disease. Since myelin is mainly composed of complex lipids synthesized by oligodendrocytes, reduced levels of galactosyl ceramide (cerebroside) and sulfatide can be found in both the grey and white matters of AD brains [89]. These compounds are the most specific lipids of myelin, decreasing in parallel with the severity of the disease and altering long before fibrillary deposits of tau protein [28, 87]. Cholesterol concentrations, another majority lipid compound of myelin sheaths, is known to decrease with the onset of cerebral atrophy [44].

Myelin proteins are also involved in relatively early stages of the disease (Braak stage I and II), in which alterations of oligodendrocytes and myelin are noted even before the onset of clinically detectable cognitive disorders [55]. The level of most myelin proteins is likewise decreased in more advanced stages of AD (Braak stages V and VI). Lowered concentrations of basic myelin protein (MBP), proteolipid (PLP) and 2’-3’ cyclic nucleotide phosphodiesterase (CNPase) are observed, specifically in several regions of the cerebral cortex.

In the field of protein markers present in patients’ CSF, there is a wide heterogeneity and variability, which confirm the impression that sporadic AD may be the consequence of multiple and varied alterations in many metabolic circuits. This reinforces the idea that the pathophysiological mechanisms leading to late AD are multifactorial and reveal a disease of great complexity [205]. Many cognitive pathologies with MCI are often accompanied in a non-specific way by the presence of inflammatory markers and proteins associated with the complement cascade in the CSF or blood of patients.

### Oligodendrocyte’s dysfunction: A major risk factor in AD and a process in the onset of the disease?

Before the appearance of amyloid and tau pathology, many forms of AD showed a breakdown of myelin due to the vulnerability of oligodendrocytes under this neurodegenerative pathology. In many cases, the loss of myelin sheaths appears to be the initiating step in the earliest stages of the disease. Extensive evidence has indicated that the breakdown of myelin is associated with AD since the vulnerability of oligodendrocytes under Alzheimer’s pathology easily induces the myelin breakdown and the loss of the myelin sheath.

Aging itself is already an important factor of myelin alterations and multiple cellular partners are involved in this process. Brain MRI often reveals signs including several hyper signal outbreaks in T2-weighted images (T2WI) with chronic cerebral hypoperfusion often associated with carotid stenosis [112]. These alterations appear more massive at the stage of MCI both in animal models and in human pathology than in established AD. In myelin abnormalities, association of oligodendrocytes’ losses with axonal alterations are commonly encountered in post-mortem patients [137]. The accumulation of Aβ peptides is considered a princeps factor in the neurodegenerative process even before the appearance of aggregates in the form of amyloid plaques [23, 75]. The response of oligodendrocytes to the presence of amyloid peptides or plaques has been the subject of several studies. During aging, the spontaneous involution of these cells is important, and their disappearance is close to 25% from the age of 50 years, this phenomenon being potentiated by the presence of the *APOE* ε4 allele in the genomic baggage of the individual [107, 132, 134]. Furthermore, the importance of increase in the expression of myelinating genes in oligodendrocytes from Alzheimer’s patients is related to the severity of the disease [64, 79]. In transgenic animals over-expressing the APP, the myelin sheath has an increased thickness and a modified architecture [54, 67, 211].

Originating mostly from the ventricular and subventricular regions of the brain, OPCs are present in the brain, even in the adult stage [36, 164, 187, 218]. OPCs control the angiogenesis of the white matter, its vascularization/oxygenation, and the myelination of axons according to spatial-temporal parameters, which contribute to their stability, functions, and integrity [96, 153]. Intimate exchanges between neurons regulate ionic homeostasis, making of real synaptic connections and activity of OPCs via numerous neurotransmitters [58, 68, 172]. These cells are also the target for several mitogens produced by neurons and trophic factors like neuroregulin 1 and brain-derived neurotrophic factors (BDNF), whose release depends on neuronal activity [77, 199]. Many studies have been conducted to explore early oligodendrocyte alterations in AD in association with changes in myelination and early symptoms of the disease. Most commonly, oligodendrocyte differentiation abnormalities are associated with disruption of oxidative stress phenomena associated with excitotoxicity, mediated by glutamatergic metabotropic receptors in large amounts in oligodendrocyte precursors [22, 137]. Other factors, such as high iron ion levels and disorders in the glutathione cycle, would accentuate the presence of free radicals, without forgetting the mitochondrial chain disorders induced by the toxicity of Aβ peptides [190]. Mitochondria pathologies are at the forefront of axonal survival for functional and metabolic exchanges with the myelin envelope [66, 219].

Myelination is directly related to the intensity of neural activity, which affects the electrical properties of axons. The toxicity of Aβ peptides proteinopathy affects immediately the whole myelin-axon, which forms a couple with multiple functional and metabolic relationships [181].

The toxicity of proteinopathies, which causes degenerations in AD, mainly concerns oligomeric Aβ peptides and hyperphosphorylated tau proteins [2, 41, 82]. Senile plaques are rarely seen in the hydrophobic white matter and does not lend itself to the aggregation of toxic oligomers. As a result, it is mainly these latter that exert toxicity on myelin. The therapeutic strategies currently developed for plaque removal seem not to display huge impact on clinical symptomatology [196]. In fact, the degree to which the therapeutic strategies for plaque removal have clinical effects remains an open question, as the reason why such therapies are not working well. For more than twenty years, therapeutic research against AD has focused on reducing the accumulation of pathological amyloid peptides and the substances studied have made it possible to achieve this goal without significant improvement in cognitive impairment in patients. The amyloid hypothesis revised in many cases is questioned, sometimes in favor of primitive alterations of the myelin envelope [61]. Oligodendrocytes are very active cells from a metabolic point of view, especially during the process of myelination or remyelination. A cellular respiratory abnormality that may be related to hypo-vascularization or ischemia may be the source of a myelination disorder [137]. Vascular pathologies affecting the white matter are common in the elderly or with symptomatologically occurring early AD [78, 109]. The human brain is largely myelinated, which may partly explain its vulnerability to neurodegeneration [192].

Oligodendrocytes are widely represented in many areas of the human CNS, especially in the neocortex, where they account for about 75% of glial cells [45]. They are considered very fragile, and their density decreases sharply in the brain of the elderly person from 50 years of age [223]. Various methods of labeling these cells have shown a severe loss of oligodendrocytes in several regions of the hippocampus, not correlated with the density of amyloid β deposits [40]. These specific depopulations probably precede the disorganization of the neural connectome that precedes the appearance of AD, and a contemporary demyelination around the outbreaks of amyloid peptide deposits [94]. These dysfunctions are strongly associated with abnormalities in lipoprotein metabolism given that the amyloid oligodendrocytes actively participate in the synthesis of cholesterol constituting synaptic contacts [120]. They secrete apolipoproteins E and J, which are severe risk factors depending on the alleles involved in the onset of AD [85]. Interestingly, the production of new oligodendrocytes seems fundamental for motor learning in mice [111].

During the development of AD, including in the early stages characterized by mild, worsening memory disorders (MCI), numerous studies have been performed to characterize the changes observable by MRI techniques in the structure and architecture of myelin. Schematically, the results obtained showed very early the existence of a reduction in cerebral myelin levels, with losses of oligodendrocytes and axons, microglial activations accompanied by dilated perivascular regions in the white matter. These studies are essentially based on the contrasts between the aqueous contents of the intra- and extra-cellular spaces at the periventricular level, comparing MCI patients and control persons. It seems that progressive ischemia with vascular and energy losses associated with the toxicity of certain proteinopathies (especially amyloidosis Aβ) alters the myelin structure very early and hinders proliferation and oligodendrocytic re-myelination [88, 137, 148].

## Adaptative immunity to myelin components in AD

### An auto-immune process for degeneration?

Many results support the existence of mutual interactions between immune processes (innate and acquired) and neurodegenerative events, especially those occurring during the incubation of AD [33, 51, 144].

Neuroinflammation phenomena are considered to pre-exist for a very long time in the brain before the onset of cellular stigmas of neurodegeneration and clinical symptomatology. Chronic inflammation, microglia activation and lymphocytic infiltration are thought to be the result of intracerebral accumulation of misfolded proteins and/or multiple exogenous attacks of various infectious agents during the individual’s lifetime [42]. Amyloid peptides and hyperphosphorylated tau are particularly involved in the inflammatory reaction and progressive onset of autoimmunity [207]. Changes in circulating cytokines as well as disorders in the cascades of the complement and clotting factors testify to changes in the immune response at the periphery [143]. Cleavage fragments of abnormal proteins, numerous glycated proteins and a large population of phosphoproteins contribute to microglial activation in the brain and alteration of many resident proteins [24]. This include myelin constituent proteins that are presented as new antigens to the immune system. Studies have shown significant accumulation of autoantibodies in the serum of patients with AD, especially directed against myelin proteins [62, 115]. IgG and IgM immunoglobulins directed against the MOG, MBP, MAG and PLP proteins are frequently present in the CSF and circulating blood [152]. This strongly suggests the involvement of the immune system in myelin alterations observed in many AD patients and in some animal models of the disease.

Since the discovery of mutations in *APP*, *PSEN1* and *PSEN2* genes, which induce familial ADs, the hypothesis of the amyloid cascade at the origin of the pathophysiology of AD remains the preferred mechanism of this type of neurodegeneration [136]. The problem is that sporadic AD does not usually present this type of mutation, although similar pathologies of Aβ peptides and tau proteins are encountered in familial and late forms of the disease [106]. The main hypothesis remains those long-term abnormalities in Aβ peptide metabolism are the starting point of tau dysfunction and a series of toxic phenomena inducing neuronal and cognitive losses. It appears that during aging, multiple mutations accumulate in the nuclear and mitochondrial DNA of neural cells that add up to the increased loss of editing and quality control of translated proteins [76, 108]. It is estimated that in the normal individual, about 20% of the proteins synthesized by ribosomes have structural and folding abnormalities and must be eliminated by the proteasome, lysosome, or resident proteases of the cell membrane [97, 157, 180]. Overloading these mechanisms leads to chronic neuroinflammation, microglial and macrophagic activation, and immune responses against abnormal non-functional proteins over the long-term [122]. Among the oligomeric peptides that accumulate in the brain and display significant cellular toxicity are Aβ peptides and particularly the peptide Aβ1–42. This accumulation most often comes from a drop in the clearance to the vascular compartment and the CSF.

The rupture of the myelin envelope appears to be an early phenomenon in the pathophysiology of AD [38, 150, 202]. In humans, the vulnerability of myelin materializes on MRI through morphological changes, thinning and hydration swelling [49, 229]. At the same time, there are elevations of tau, phosphotau, soluble APPβ (sAPPβ) peptides and Aβ1–42 peptides. The latter peptide has a high toxicity to myelin in oligodendrocyte cultures and in animal models of familial AD (e.g. 5XFAD mice), where morphological alterations of myelin are the first pathological stigmas to appear in animals at 1 month of age [67]. The mechanisms of toxicity of peptides Aβ are still the subject of speculation; it seems that the oligomeric Aβ peptides are the main culprits of this toxicity [75]. Several cellular receptors (glutamates, ephrin’s, adrenergic, cholinergic, and immunoglobulins) bind oligomeric Aβ peptides and could mediate the toxicity of amyloid oligomers during the years of incubation of the disease [171, 215].

### Multi-proteinopathies are associated with aging brains

During lifetime, mutations accumulate in the post-mitotic cells of neurons due to non-replication of DNA, but also within mitochondrial DNA [86, 95]. This results in harmful mitochondriopathies for neuronal survival, as well as increased production of misfolded and non-functional proteins. Deficiencies in quality control and protein structure editing also contribute to the intra-brain accumulation of protease-resistant hydrophobic deposits with intrinsic toxicity [1, 151]. These chronic accumulations lead to long-term inflammatory and phagocytic reactions, as well as immune responses accompanied by infiltration of immunocompetent cells. Gradually, an amyloid reaction develops, in which peptides Aβ participates largely because of their cerebral accumulation. The elimination of these peptides from the brain is largely conditioned by the effectiveness of the enzymes that degrade them and allow their clearance [222]. The peptide Aβ1–42 is particularly toxic to myelin sheaths, axonal and synaptic endings that finally degenerate [75, 137]. In several transgenic models of familial forms of Alzheimer’s, the first stigmas of the disease result in morphological abnormalities of myelin sheaths, in the form of edema and thinning of the envelope surrounding myelinated axons. At the same time, disturbances in animal behavior appear manifesting as reduced anxiety manifestations and reduction of memory and spatial recognition [56, 57].

**Fig. 4:**
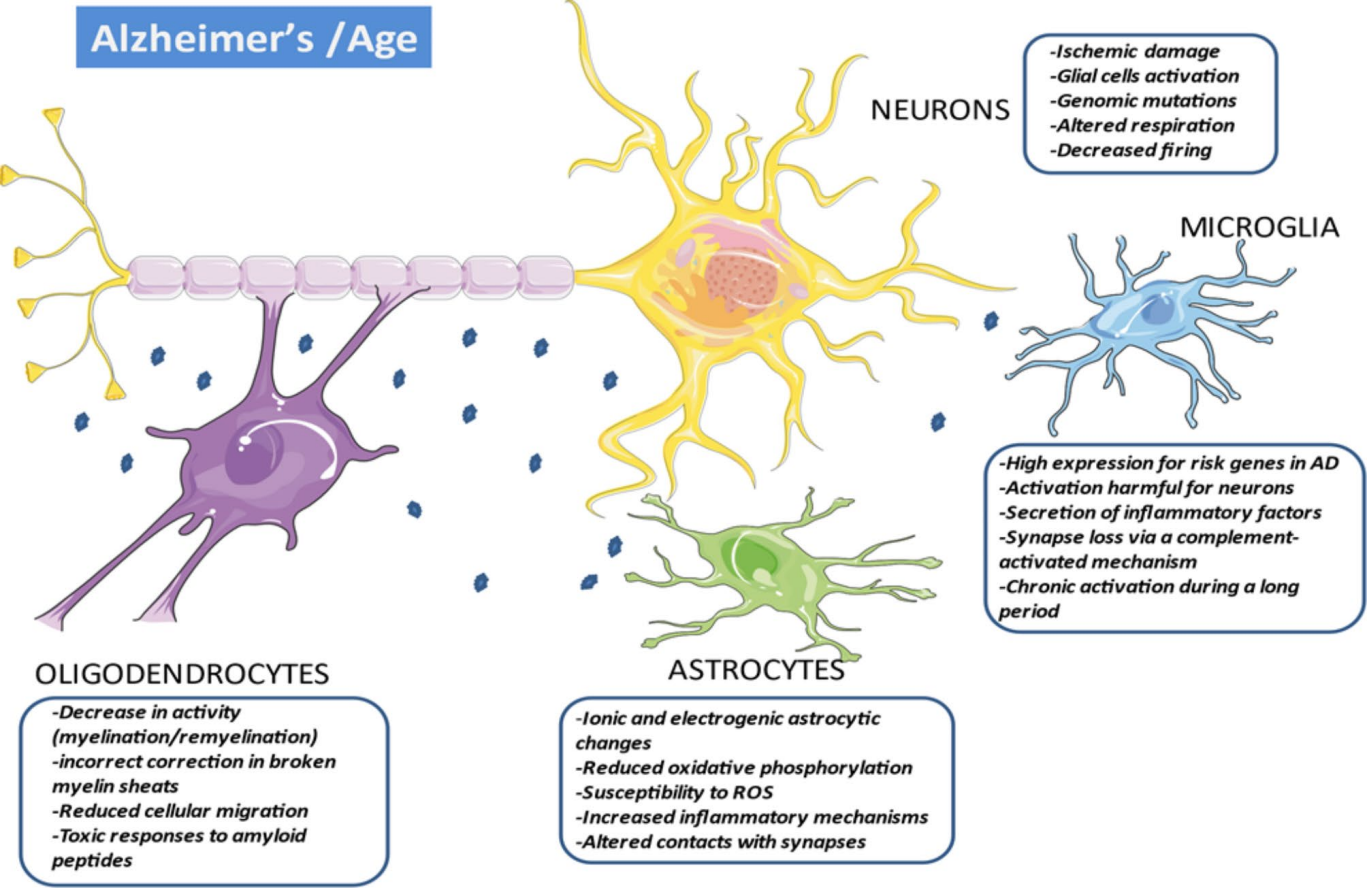
**Multiple Alzheimer’s disease etiologies and many cellular partners.** Deleterious proteinopathies (in the first-place amyloid peptides) are to be integrated into the complex cellular environment of the brain. These multiple cellular elements participate in progressive multi-focal neuro-axonal degeneration leading to the irreversible symptomatology of AD. This is expressed when the toxic peptide removal systems are overwhelmed, which appears only after a long incubation period. Altered neurons express phases of hypo- and hyperexcitability with deficits in axonal transport and synaptic activity that affects myelination/remyelination activity and oligodendrocyte trophism. These are very vulnerable cells whose density decreases sharply with age. There seems to be a link between the intensity of neuronal involvement and the extent of demyelination. This is strongly accentuated in AD in which remyelination processes seem deficient. The activation of astrocytes participates in the elimination of deficient neurons and synapses. They actively participate in the elimination of abnormal proteins and inflammation processes, in the same way that the activation of microglia facilitates the phagocytosis of cellular debris. In the same way, these cells participate in the activation of the innate immune responses, the activation of the complement and the secretion of inflammatory cytokines. ***Abbreviations***: *AD, Alzheimer’s disease; ROS, reactive oxygen species*

Depending on the individual, brain aging does not occur unequivocally but depends on multiple factors related to specific genes and environmental situations (Fig. 4) [176]. The accumulation of mutations in the nuclear DNA of post-mitotic cells and mitochondrial DNA induce deleterious mitochondriopathies [121, 186], promote the production of abnormal proteins, impair respiratory and energy functions, and amplify cellular and oxidative stress [91]. Toxicity of abnormal oligomers seems to be the result of their misfolded nature, which exposes hydrophobic residues leading to aggregation and abnormal interactions with a large range of cellular components [4]. Membranes like myelin constituted mainly by complex hydrophobic lipids could be an important target for amyloid oligomers for direct interactions andymes modifications inducing inflammatory and immune responses.

Several evidence from studies of the population of abnormal proteins in the CSF showed that abnormal proteins in CSF represent a picture close to that of abnormal proteins in the brain [11]. This methodology can provide information on the biochemical and metabolic changes that occur in the CNS of patients with neurodegeneration. CSF amyloid peptides and tau proteins are used for the diagnosis and evolution of AD [154]. Aβ1–42 peptides correlation has been described with several CSF proteins belonging to the endocannabinoid and the somatostatin systems [71] with the latter regulating the proteolytic degradation of the amyloid peptide. The presence of other proteins has been linked to the degradation of the myelin [170].

The quality control processes of in vivo newly formed proteins and the elimination of abnormal proteins are phenomena with growing alterations with age [92, 138]. This results in the cellular and extra-cellular accumulation of an increasing number of non-functional proteins that tend to form hydrophobic aggregates [117]. At the brain level, these toxic aggregates induce significant cellular and functional losses that are the basis of many neurodegenerative diseases [14]. In addition to the amyloid peptides and tau protein that are the canonical proteins of early AD and whose toxic deposits in brain tissue are the basis of mechanistic theories of neurodegeneration, it has been shown that a wide range of protein aggregates from other sources exist in the brain of elderly patients displaying a cognitive impairment or at first stages of AD [162]. Among the proteins significantly altered compared to controls, many are found in the biochemical cascade of glycolysis that primarily feeds cellular energy and whose intensity decreases with age, even faster in patients with AD [135]. Other strategic proteins form larger insoluble aggregates depending on symptomatologic impairment. These include glucose 6 phosphate isomerase creatine kinase B, certain forms of adenylate cyclase and calcium/calmodulin protein kinase 2. This list is not exhaustive but reflects the importance of metabolic and functional disorders that develop over time in the brains of patients with mild cognitive impairments that worsen in AD [91].

It could be speculated that the accumulation of misfolded proteins during old age in multiple regions of the brain alters mitochondrial and metabolic functions, saturates the processes of cleaning and elimination of senescent cells, and slows down the neurogenesis that persists in the older brain [20, 80]. Inflammatory vasculitis, hypoxia, and oxidative stress due to the accumulation of non-functional deleterious proteins are considered the primary factors in myelin envelope impairment. The decrease in electrical and metabolic activity of axons contributes to the decrease in myelin density that surrounds them leading to its gradual dislocation. The toxicity of Aβ peptides has been demonstrated in vitro against neurons, endothelial cells, astrocytes, vascular smooth muscle cells and oligodendrocytes [224]. Aβ peptides cytotoxicity might involve the susceptibility of oligodendrocytes to oxidative stress because of its low content of reduced glutathione and high concentration of iron [190]. The Aβ peptides activation of the neutral sphingomyelinase-ceramide pathway has been reported to induce oligodendrocyte death [101]. In addition, inhibition of neutral sphingomyelinase 2 in these cells reduces their ceramide content and favor the myelination process by improving the quality of myelin structure [221].

## Microbiome and myelin dysregulation in the neurodegenerative brain

### A role for the gut-brain axis and for hepatic metabolism

Some arguments favor a view of AD as a disease that is not limited to the CNS alone but reflects multi-organ dysfunctions that contribute to or influence brain neurodegeneration [200]. Multiple proteinopathies, including the Aβ cascade, may come from peripheral organs that no longer metabolize abnormal proteins properly and allow their dissemination through a permeable BBB. Abnormal communication between various compartments of amyloid proteins can contribute to altering brain disease. Chronic peripheral metabolic abnormalities are suspected to participate or to worse neurodegeneration. In this regard, intestinal metabolism is often questioned.

The population of microorganisms of the gut microbiota constitutes a true symbiotic organ that has a great inter-individual heterogeneity due to many intrinsic and extrinsic factors dependent on genetic, medication (e.g. antibiotics), physical and hormonal activity, and infectious factors [185]. The composition of the microbiome changes with age and the reactivity of the immune system [129]. An active exchange via the bloodstream and intestinal innervation between the microbiome and the nervous system exist, whose influence is important during the neurogenesis, the molecular organization of the connectome, and the variations of CNS myelination. These phenomena are especially different during periods of brain development or during aging [228].

**Fig. 5:**
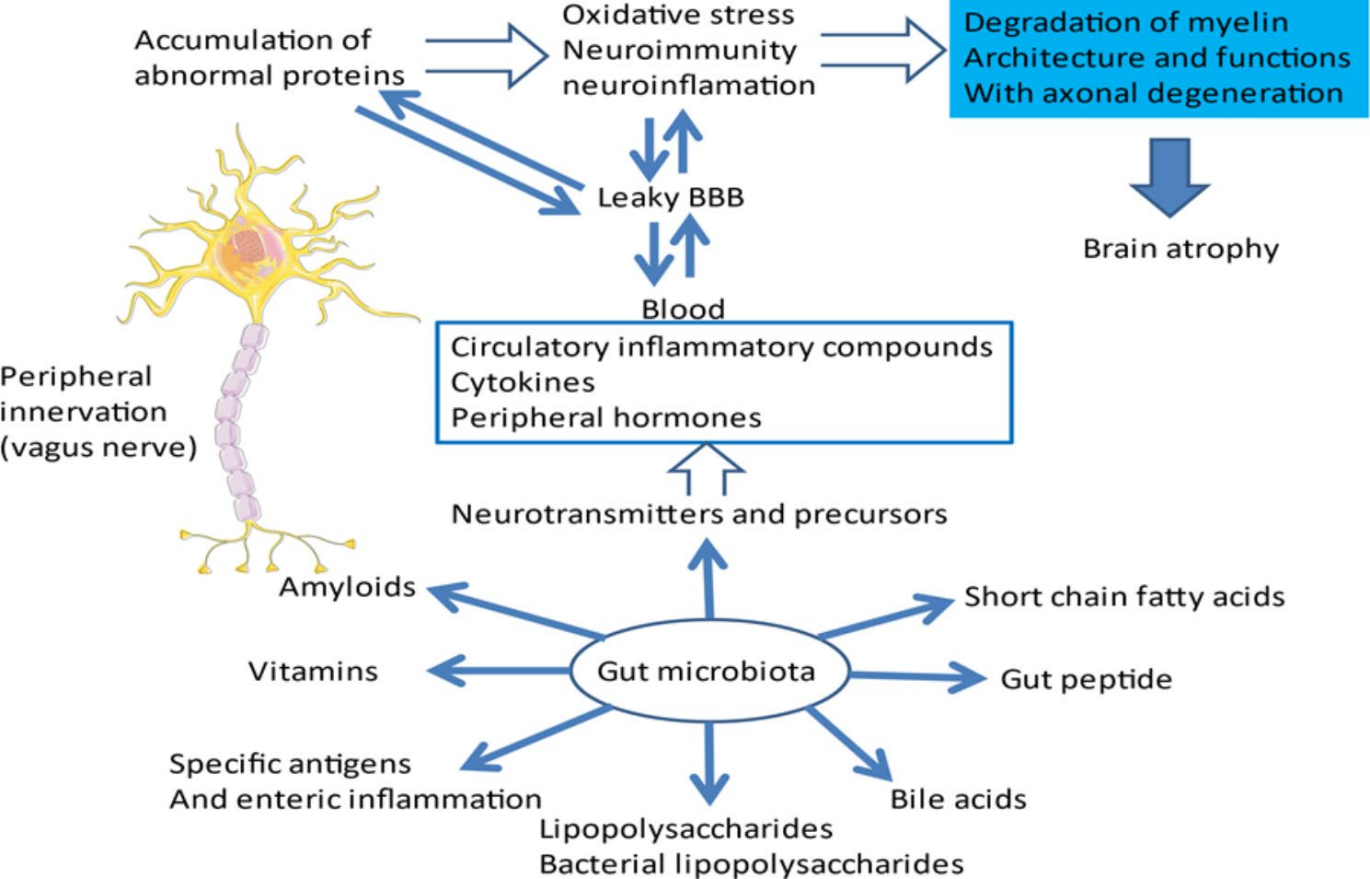
**Multiple communication system that includes neural, immune, endocrine, and metabolic pathways lead to degeneration.** Continuous fluctuation of the microbiota due to the environment constantly influences the inflammatory, immune, and metabolic responses of the CNS [110]. With age, the permeability of intestinal and BBB is often impaired [84, 161]. The gut microbiota metabolizes and release many growths, metabolic and inflammatory factors which could penetrate the brain via the circulating blood. These substances contribute to increase the inflammatory, immune, and oxidative phenomena that exist in the elderly brain due to the accumulation over time of many abnormal proteins due to their hydrophobic conformation. The very likely origin of these malformed proteins is found in the accumulation during senescence of many mutations in post-mitotic cells that are neurons [9, 76]. In addition, the role of epigenetic dysregulation of gene expression induced by aging or abnormal environmental stimulation is also considered to be an important factor in neurodegeneration and cognitive alterations [125]. ***Abbreviations***: *BBB, blood-brain barrier; CNS, central nervous system*

In various transgenic models of familial AD, disturbances in the composition and diversity of the intestinal microbiome compared to healthy animals are observed [174]. In humans, it has also been described qualitative and quantitative changes in the population of intestinal bacteria in patients with cognitive disorders associated with cerebral amyloidosis [59, 118]. These disturbances can be the source of chronic neuroinflammation targeting several organs including the brain and a decrease in the immune response inducing neurodegeneration over the long term [26, 74]. These phenomena are increased by the leaky permeability of the BBB as a function of age, allowing the passage at the cerebral level of many toxics present at the periphery (Fig. 5) [213].

The problems of pathological cerebral aging are probably the result either of the evolution of protein targets at the central level, or the modification of a peripheral immune response [60, 182], the two mechanisms can combine over time to lead to an autoimmune alteration colonizing the CNS and involving, in the first place, the components of myelin. Many environmental factors can promote this chronic process by perpetuating the homeostasis of the intestinal flora and at the origin of certain metabolic and cytotoxic disorders [15, 90]. This primarily affect oligodendrocytes, which are fragile cells of the CNS, and which adapt their functions throughout the life of the individual. Many factors contribute to the activity of oligodendrocytes, intrinsic and environmental factors that modify the status of the epigenome [165]. Among these factors, the composition and activity of the microbiome plays a reweighting role and interferes with the spatio-temporal character of myelination in the brain. In general, the relationships between the intestinal sphere and the brain are of primary importance for myelination. This sphere includes not only the intestinal epithelium, but also hepatic metabolism, sympathetic and parasympathetic nerve activity, endocrine, and cytokine secretions and metabolites of microbial origin [46]. The microbiota has an important role in the regulation of myelin plasticity as the existence of hyper-myelinated axons has been demonstrated in germ-free mice or treated chronically with antibiotics [72]. This abnormality could be a consequence of neuronal hyperactivity in certain regions of the brain of these mice, such as the amygdala or the prefrontal cortex. The development of myelinating oligodendrocytes is controlled by a set of transcription factors (Sox 10 and Myrf for example) that drive the steps of myelination and re-myelination [7]. The anomalies of these phenomena alter certain brain functions, those concerning cognitive functions. Restoring a normal microbiome in germ-free mice greatly improves their social and executive performance [173, 189, 191].

It is now recognized that disorders intestinal physiology can influence the risk of Alzheimer’s and its rate of progression. Deposits of aggregate of Aβ peptides at the intestinal level have been detected in AD patients [74], but most of the results involving the intestinal sphere and the progression of AD have been obtained in animal models. The ratios between Firmicutes and Bacteroidetes are considered strategic in the composition of the human intestinal microbiota [168]. The fecal microbiota is the product of a very complex and diverse ecosystem, and its composition can modify the accumulation of intestinal APP in the early phases of AD [29, 119]. In transgenic APP/PS1 animals, an increase in Aβ peptides levels have been observed in the CNS in relation to changes in the intestinal flora, accompanied by disorders of spatial memory [225]. Oligodendrocytes and myelin sheaths may be the first to be affected by these deleterious deposits. A parallel can be observed between the myelin alterations observed in AD and during normal aging in the elderly. In the latter case, the installation of progressive ischemia could be the cause of this demyelination [137, 177]. The lesions often appear disseminated with a predilection for intracortical axons of small diameters that are myelinated late during development. Myelin dystrophies lead to axonal alterations and neuronal death with different rate in individuals [163].

## Conclusion and perspectives

To conclude, myelin damage and its several possible outcomes (Table 1) is one of the early lesions observed in many clinical forms of AD. Even though many differences exist in the presentations and structural alterations between multiple sclerosis and AD, neurodegenerative alterations between both pathologies have common etiologies and mechanisms [113]: long-standing inflammatory disorders, some autoimmune reactions, cognitive impairments, and mitochondrial alterations [104, 184]. Amyloid disorders are not absent from the pathophysiology of multiple sclerosis and Aβ peptides levels are generally lower in the CSF of patients with multiple sclerosis who have cognitive impairment [93]. The accumulation of APP in the brain of these patients appears parallel to the worsening of symptomatology and dynamic processes of demyelination/remyelination [25]. These parallels remain hypotheses at present, but there are indications that some mechanistic similarities exist.

**Table 1:** Examples of different outcomes of myelin damages

Myelin modifications	References
Demyelination (as evidenced, for example, by decreased myelin water fraction)	[18, 48, 83, 94, 137, 177]
Rupture of the myelin envelope	[38, 150, 202]
Myelin reshuffle	[16, 47, 165, 179]
Defect in myelin biosynthesis (loss of ceramide synthase 2 activity)	[32]
Down-regulation of myelination network	[3]
Morphological abnormalities of myelin sheaths, in the form of edema and thinning of the envelope surrounding myelinated axons	[49, 137, 229]
Myelin degeneration -> driving cognitive and motor impairment	[27, 153]
Changes of myelin organization (q-Space myelin map imaging)	[148]
Myelin instability	[35, 203]
Myelin damage in cortical gray matter (Western blot quantification of MBP and dMBP)	[227]
Decrease of myelin density (multi-echo T2 relaxation time technique)	[99]

Current mechanistic hypothesis favors long-term dysfunctions in the proteolysis of APP and in the accumulation of hydrophobic Aβ peptides with multiple toxicities. These lead to inflammatory, oxidative, and immune reactions leading to massive cellular apoptosis accompanied by post-translational modifications on target proteins inducing profound functional alterations in brain cells activities. It seems possible that a multiplicity of mutations and epigenetic alterations of neuronal genomes, associated with intrinsic or extrinsic predisposing factors, generate metabolic and inflammatory alterations over the long term, inducing a multiplicity of phenotypic and clinical presentations involving secondarily multiple deleterious proteinopathies, including amyloidosis of Aβ types.

## Data Availability

Not applicable.
